# Non-recurrent inferior laryngeal nerve: case report and review of the literature

**Published:** 2014

**Authors:** R Iorgulescu, I Bistriceanu, D Badanoiu, C Calin, C Capatana, N Iordache

**Affiliations:** * Emergency Clinical Hospital “Sf. Ioan” Bucharest; ** National Institute of Endocrinology “CI Parhon” Bucharest

**Keywords:** total thyroidectomy, inferior laryngeal nerve, non-recurrent

## Abstract

Total thyroidectomy is nowadays the operation of choice in the majority of endocrine surgery centers, whether the pathology is benign or malignant. To obtain good results, a thorough knowledge of local anatomy and a profound respect for hemostasis are necessary. Routine, at least visual, identification of the inferior laryngeal nerve (ILN) is considered gold standard and is strongly recommended. Surgeons are generally aware of the variations the nerve can have, especially on the right side. Although very rare, one such variation, with possible great impact on outcomes, is the non-recurrent route of the nerve. We present the case of a middle-aged woman with a multinodular goiter scheduled for elective surgery. During total thyroidectomy, on the right side, we were not able to find the inferior laryngeal nerve in its usual position, using the customary anatomical landmarks. Instead, we encountered it emerging directly from the right vagus nerve, at a rather right angle and entering the larynx as a unique non-bifurcating nerve. Thus, it could be spared from any injury and protected, although it could have been easily confounded with a vascular structure, given its transverse course.We think it is never overmuch to repeat that the routine identification and exposure of the inferior laryngeal nerve is a must for the thyroid surgeon in order to safely preserve its integrity.

## Introduction

In surgery in general and particularly in thyroid surgery, a thorough knowledge of local anatomy is of paramount importance for obtaining outcomes of expected standards.

Nowadays, a large majority of thyroid surgeons agree that, whether the thyroid pathology is benign or malignant, the operation of choice has to be total thyroidectomy [**1**, **2**, **3**]. Thyroidectomy is carried out in narrow spaces, full of anatomical structures, some of which can have great variability. These variations can be encountered any time and hence have to be born in mind by the operating team all the time.

In this respect, the inferior laryngeal nerve is still considered a remarkable element, somehow unique from a point of view, although our knowledge of the anatomy and physiology of this nerve began some 2000 years ago. It not only has a great variability regarding its thickness, its route (especially in relation to the inferior thyroid artery and its branches) and its distal ramification, but also can sometimes be so atypical as to be non-recurrent at all.

## Case presentation

A 46 years old woman was admitted for elective surgery with the diagnosis of multinodular goiter. The specialist endocrinologist established the indication for surgical treatment and the present euthyroid status of the patient.

The clinical exam revealed a rubberlike, rather irregular and big goiter, developed predominately on the left side, apparently going beyond the anterior margin of the sternomastoid, but with no signs or symptoms of local compression. No notable co-morbidities were present.

The operating team consisted of one consultant with special interest in endocrine surgery and medium – high volume of thyroid and parathyroid cases (50 – 60 operations per year) and 2 registrars.

**Fig. 1:**
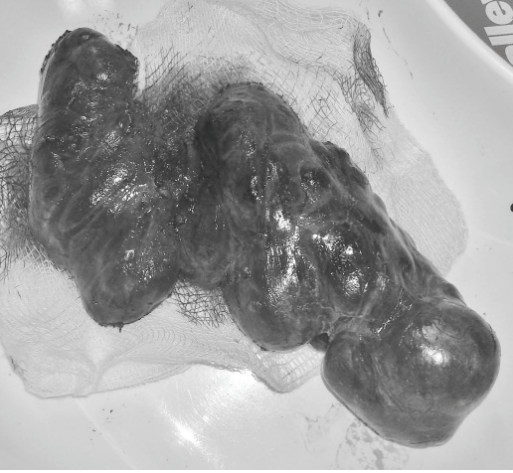
Total thyroidectomy for a multinodular goiter with the left lobe significantly bigger

**Fig. 2:**
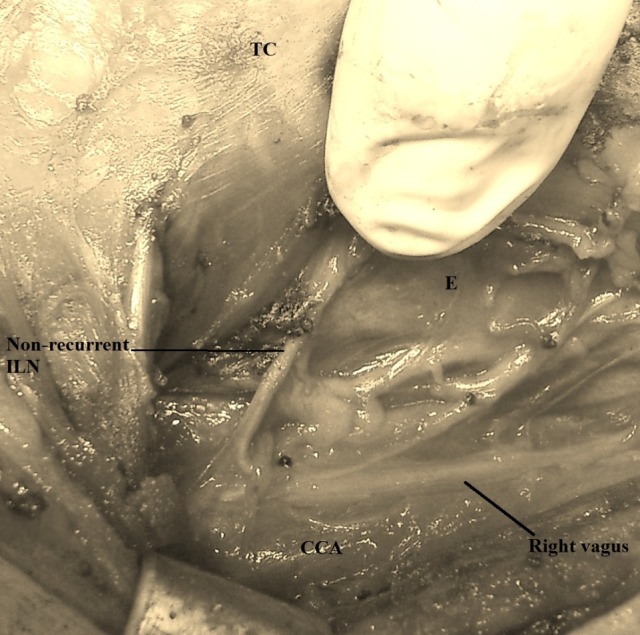
A transverse nervous structure emerging from the right vagus – non-recurrent inferior laryngeal nerve (ILN); the thyroid have been excised; CCA – common carotid artery; E – esophagus; TC – thyroid cartilage

Under general anesthesia, the surgical team proceeded to total thyroidectomy with routine identification of the inferior laryngeal nerve and, at least, superior parathyroid glands, with the vascular cutting and sealing done with Harmonic Ace CS14S. Although the left lobe was significantly bigger (**Fig. 1**), the dissection and identification of the recurrent laryngeal nerve were straightforward. On the right,we first mobilized the lobe anteriorly and medially by severing the middle thyroid vein and the vessels of the upper pole. We identified the inferior thyroid artery and its branches and, as we usually do, we commenced the search of the ILN, first cranially and then caudally, being unable to find any nervous structure resembling ILN in the tracheo-esophageal groove. Therefore, we thought of the possibility of the ILN being non-recurrent, identified the vagus nerve and followed it cranially. In this way, we encountered a transverse structure emerging from the vagus, approximately at the level of the cricothyroid junction and entering the larynx at the lower margin of the cricothyroid muscle. We carefully dissected its entire length and preserved this structure (**Fig. 2**) that proved to be a non-recurrent ILN.

The postoperative course was uneventful, without any sign of hypocalcaemia, without dysphonia and the repeat indirect laryngoscopic exam was normal.

## Discussion

The anatomical variation on the ILN, especially on the right side, is abundantly documented and well known by the endocrine surgeon.

A very rare such variant, almost exclusively on the right side,is the non-recurrent inferior laryngeal nerve (NRILN). In was first described by G.W. Stedman in 1823 [**4**, **5**] during a cervico-thoracic dissection made at the Royal Academy of Copenhagen, when he also noticed the arterial anomaly accompanying it. Most authors consider that it appears with a frequency between 0,3% and 1,6% [**5**], although a French group from Lille, in a study published in 2013, estimates a much higher incidence, up to 6%, with systematic use of intraoperative neuromonitoring (IONM) during thyroid surgery [**6**].

**Fig. 3:**
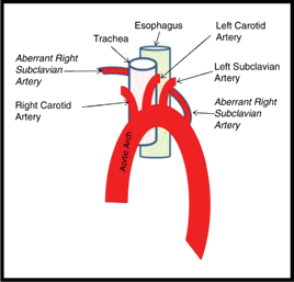
Aberrant right subclavian artery emerging from the aortic arch after the left subclavian (7)

The anomaly is justified by a developmental embryological error of the aortic arch and its main branches that makes the right subclavian artery to emerge distally, after the left subclavian artery (**Fig. 3**). In its way towards the right arm, the right subclavian usually passes behind the esophagus (80%), but can also pass between the trachea and the esophagus (15%) and even in front of the trachea (5%) [**8**]. The anomaly is usually asymptomatic. When it manifest itself, in less than 10% of cases [**9**], it causes swallowing difficulties by esophageal extrinsic compression, a dysphagia named “lusoria”, as is also named the aberrant right subclavian artery (from Latin “lusus nature” meaning “a freak of nature”). It is almost impossible to differentiate it clinically from other causes of dysphagia, especially from those determined by the enlargement of the thyroid gland [**10**].

The situation in which there is a NRILN without any vascular anomaly is also described in the surgical literature and no appropriate explanation can be offered [**11**]. Some authors consider it a so-called “false” NRILN, being in fact an anastomotic ramus between the sympathetic cervical trunk and a very thin recurrent ILN [**12**]. In the present patient, we could not find a coexisting recurrent ILN.

The coexistence of a second, thin, recurrent ILN is a point of debate and not all authors agree that such an entity is genuine. What is worth mentioning is the opinion that the finding of a very thin right recurrent ILN should put the surgeon on guard and make him search for a larger non-recurrent trunk [**13**, **14**].

A NRILN can be present on the left side only in case of dextrocardia or situs inversus and occurs in some 0,04% of cases [**13**].

We found at least the following classifications that take into consideration the level at which the NRILN derives from the vagus and its trajectory towards the larynx.

One of them considers that there are three types on NRILN [**4**]:

- Type IA, the nerve has a linear course at the level of the upper thyroid pole

- Type IB (most frequent), the nerve goes transversally at the level of the thyroid isthmus

- Type II, the nerve takes a downward curve, reaching the lower pole of the right lobe.

Other authors also distinguish three types of NRILN [**5**]:

- Type 1, that runs with the superior thyroid vascular pedicle

- Type 2A, that runs transversally, parallel to the inferior thyroid artery and over its trunk

- Type 2B, that runs transversally, but under the trunk or between the branches of the inferior thyroid artery.

Moreover, another classification estimates that there are two main types of trajectory of the NRILN [**8**]:

- The high type (type I), taking a short route, perpendicularly from the vagus to the laryngo-tracheal junction

- The low type (type II), with variable takeoff from the vagus, which describes a concavity oriented superiorly and externally, and then reaches the tracheoesophageal groove.

As it can be seen in (**Fig. 2**), in our patient, the NRILN had a short, linear route, approximately at the level of the crico-thyroid junction.

More important than any classification is that the positive diagnosis can almost never be established preoperatively, a fact that emphasizes the importance of the surgical technique and intraoperative detection.

Some say that barium swallow could reliable detect a “lusoria” retroesophageal artery, should it be suspected, by the indentation it leaves on the posterior esophageal wall, resulting in a “bayonet” image [**13**]. The endoscopic exam can confirm the radiologic finding, demonstrating the pulsations transmitted on the posterior wall of the esophagus [**5**].

There is a multitude of imaging techniques and examinations of high performance that can highlight the vascular anomaly and therefore make the surgeon think of a probable NRILN. The postoperative CT, angio-CT, angio-MR employment is rather futile and has only a documental and academic value. What would be of practical importance is, as we have already mentioned, the preoperative detection and, in this respect, we think that ultrasonography can be a solution. There are some reports in the literature that emphasize the prognostic value of this examination.

Huang and Wu consider the absence of innominate artery (brachiocephalic trunk) on the right at neck ultrasonographyprognosticative for a NRILN, with a positive predictive value of 84.6% and a 0.47% incidence of right NRILN in Chinese population [**15**].

Yetisir F. et al regard neck echography as a simple and highly reliable method in preoperative assessment, with 100% accuracy in detecting the absence of innominate artery on the right and thus predicting the presence of a NRILN [**16**]. This corresponds to the findings of Iacobone et al. who also credit neck sonography with an accuracy of 100% in correctly predicting a NRILN [**17**].

IONM was mentioned earlier as having the potential to detect the non-recurrent ILN with a higher frequency than usual. Its routine use in a strictly standardized manner, with well-defined distal and proximal vagal nerve stimulation points, provides reliable electrophysiological intraoperative verification of the presence and early identification of a non-recurrent ILN [**18**].

Given its rarity and variable origin from the vagus, it is evident that a NRILN is by far more prone to intraoperative injury than a recurrent one. In such cases, the permanent nerve lesions with vocal chord paralysis may have a frequency up till 12.9% [**5**], some five to six times more that the usual incidence of such a complication in thyroid surgery.

Consequently,the intraoperative identification and exposure of ILN is a compulsory security measure at every thyroidectomy. A prerequisite of this is a sound knowledge of local anatomical landmarks. The existence of NRILN may be an argument in favor of an early identification of the RLN, caudally, in the tracheoesophageal groove and following it upward, compared to the initial identification near its laryngeal entry point and following it downward, although the last technique is considered safer and is credited with a lower incidence of postoperative hypoparathyroidism [**19**].

Some surgeons deem that a vagus trunk situated medially to the common carotid artery is a common observation among those with NRILN [**20**]. Other surgeons go so far as to postulate that “with the exception of the middle thyroid veins, noanatomical structures passing medially from the carotidsheath should be divided or sectioned until the recurrentlaryngeal nerve has been identified” [**8**].

## Conclusion

The best way to avoid complication is prevention. Therefore, in order to stay away from any operative injury of the ILN, we have to routinely look for it, expose and protect it, constantly bearing in mind all its possible variations. The NRILN is one of them, very rare indeed, so difficult to diagnose preoperatively and so very prone to being injured. Its existence is a strong and supplementary argument for systematic dissection of ILN in thyroid and parathyroid surgery, in an orderly manner, using an adequate surgical technique.

**The authors have no conflicts of interest to disclose.**
